# Supplementary material for: Response of Pasture Nitrogen Fertilization on Greenhouse Gas Emission and Net Protein Contribution of Nellore Young Bulls
Source: Animals (Basel). 2022 Nov 16;12(22):3173. doi: 10.3390/ani12223173 (PMC9686958; doi:10.3390/ani12223173)
Supplement: Supplementary file 1 [file animals-12-03173-s001.zip › animals-1943765-supplementary.pdf]

Table S1. Weather data during experimental field data collection.

Month/year	Monthly Weather Data				
	Tmax (°C)	Tmin (°C)	Tmed (°C)	Rainfall (mm)	ND
Dec/2018	32.0	20.2	25.2	88.2	16
Jan/2019	32.7	20.9	26.1	148.1	11
Feb/2019	30.9	20.4	24.4	282.6	17
Mar/2019	31.0	20.1	24.5	115.2	12
Apr/2019	30.6	19.0	23.9	97.6	6
May/2019	29.1	16.4	21.9	24.2	4
Jun/2019	27.5	14.0	20.7	11.5	2
Jul/2019	27.2	12.2	19.7	9.3	2
Aug/2019	29.2	14.1	21.7	12.8	3
Sep/2019	33.1	18.0	25.5	79.8	6

Pressure: atmospheric pressure; Tmax: maximum temperature; Tmin: minimum temperature; Tmed: average temperature; ND: number of rainy days.
Data obtained from the agroclimatological station of the Faculty of Agricultural and Veterinary Sciences, Unesp, Jaboticabal campus.

Table S2. Chemical composition of the mineral mix offered from December to March, and the supplement supplied from April to June, during background phase.

Variable[1]	Mineral Mix	Supplement
**Ingredients, % of DM**		
Cotton seed meal	-	37
Defatted corn germ	-	21.9
Urea	-	4.6
Mineral mix	100	36.5
**Chemical composition, % of DM**		
Calcium	14.5	13
Phosphorus	8	2
Magnesium	1	0.2
Sulfur	4	2
Sodium	13	3
Crude Protein	-	32.4
NDF_{om}	-	14.5

[1] NDF_{om} = neutral detergent fiber exclusive of ash.

Table S3. Ingredients and chemical composition of the supplement, forage, and total mixed ration (TMR) of Nellore cattle during the finishing phase under different production systems.

Variable	Production Systems [1]			
	P0+PS		P75N+F [3]	P150N+F [3]
	Supplement	Pasture [2]		
**Ingredients, % of DM** [3]				
Sorghum silage	-	-	20.0	20.0
Defatted corn germ	94.4	-	75.5	75.5
Urea	1.8	-	1.5	1.5
Mineral mix	3.8	-	3.0	3.0
**Chemical composition** [4]				
DM, % as fed basis	85.5	-	74.1	74.1
OM, % of DM	93.4	92.6	90.5	90.5
CP, % of DM	17.0	7.3	15.8	15.8
NDF_{om}, % of DM	25.7	57.5	32.1	32.1

[1] P0+PS: managed pasture without N fertilization during background and fed on pasture with high supplementation during finishing; P75N+F: managed pasture with fertilization of 75 kg N ha^{-1} $year^{-1}$ during background and fed a total mixed ration (TMR) on feedlot during finishing; and P150N+F: managed pasture with fertilization of 150 kg N ha^{-1} $year^{-1}$ during background and fed a TMR on feedlot during finishing.
[2] Hand- plucked forage sampled in August 2019.
[3] Composition of Total mixed ration.
[4] DM = dry matter; OM = organic matter, CP= crude protein; NDF= neutral detergent fiber exclusive of ash.

Table S4. Characteristics of Marandu palisade grass (*Urochloa brizantha* Hochst ex A. Rich Stapf cv. Marandu) pastures of different production systems with increasing levels of N fertilization during background (wet season).

Pasture [1]	Production System [1]		
	P0+PS	P75N+F	Intensive
**Characteristics** [2]			
Herbage mass (10^3 kg DM ha^{-1})	4.6 ± 1.2	5.3 ± 1.58	5.0 ± 1.1
Green leaves (%)	24.5 ± 3.8	30 ± 6.2	33.1 ± 5.8
Stem + sheath (%)	16.2 ± 6.0	25.1 ± 8.9	28.6 ± 5.5
Dead material (%)	59.3 ± 7.5	44.9 ± 13.4	38.3 ± 7.6
Forage allowance (kg DM kg^{-1} BW)	3.0 ± 0.9	1.9 ± 0.74	1.6 ± 0.4
Leaf allowance (kg DM kg^{-1} BW)	0.78 ± 0.2	0.59 ± 0.25	0.54 ± 0.1
**Chemical composition** [3]			
OM (% of DM)	91.4 ± 0.4	91.8 ± 0.3	92.4 ± 0.5
CP (% of DM)	11.0 ± 0.3	14.7 ± 0.4	17.3 ± 0.5
NDF_{om} (% of DM)	47.8 ± 8.9	48.9 ± 4.3	47.5 ± 1.6
ADF (% of DM)	21.5 ± 4.7	19.9 ± 3.6	21.6 ± 2.6
iNDF (% of DM)	19.5 ± 2.2	17.2 ± 0.7	16.9 ± 2.6
GE (MJ kg^{-1} DM)	17.0 ± 0.1	17.3 ± 0.1	17.6 ± 0.1

[1] P0+PS: managed pasture without N fertilization during background and fed on pasture with high supplementation during finishing; P75N+F: managed pasture with fertilization of 75 kg N ha^{-1} $year^{-1}$ during background and fed a total mixed ration (TMR) on feedlot during finishing; and P150N+F: managed pasture with fertilization of 150 kg N ha^{-1} $year^{-1}$ during background and fed a TMR on feedlot during finishing.
[2] Means from January to May 2019, ± standard deviation.
[3] Hand plucked forage sampled in March 2019. BW = body weight; OM= organic matter; DM = dry matter; CP= crude protein; NDF_{om} = neutral detergent fiber exclusive of ash; iNDF = indigestible NDF; GE = gross energy.
